# Do sociodemographic risk profiles for adolescents engaging in weekly e-cigarette, cigarette, and dual product use differ?

**DOI:** 10.1186/s12889-024-18813-2

**Published:** 2024-06-10

**Authors:** Katelyn Battista, Karen A Patte, Terrance J Wade, Adam G. Cole, Tara Elton-Marshall, Kristen M Lucibello, William Pickett, Scott T Leatherdale

**Affiliations:** 1 School of Public Health Sciences, University of Waterloo, 200 University Avenue West, Waterloo, Ontario N2L 3G1 Canada; 2 Department of Health Sciences, Brock University, 1812 Sir Isaac Brock Way, St. Catharines, Ontario L2S 3A1 Canada; 3 Faculty of Health Sciences, Ontario Tech University, 2000 Simcoe Street North, Oshawa, Ontario L1G 0C5 Canada; 4 School of Epidemiology and Public Health, University of Ottawa, 75 Laurier Avenue, Ottawa, Ontario K1N 6N5 Canada; 5 Department of Public Health Sciences, Queen’s University, 99 University Avenue, Kingston, Ontario K7L 3N6 Canada

**Keywords:** Youth health, Adolescent, Vaping, E-cigarettes, Tobacco use, Nicotine, Decision trees, Classification, Risk-taking

## Abstract

**Background:**

E-cigarette use represents a contemporary mode of nicotine product use that may be changing the risk profile of participating adolescents. Understanding differences in sociodemographic characteristics of adolescents engaging in contemporary e-cigarette use and traditional cigarette use is important for effectively developing and targeting public health intervention programs. The objective of this study was to identify and compare sociodemographic risk profiles for exclusive e-cigarette use and dual-product use among a large sample of Canadian youth.

**Methods:**

A survey of 46,666 secondary school students in the 2021-22 wave of the COMPASS study measured frequency of past month e-cigarette and cigarette use as well as age, sex, gender, racial or ethnic background, spending money, relative family affluence, and having one’s own bedroom. Rates of cigarette-only, e-cigarette-only, and dual product use were calculated, and separate classification trees were run using the CART algorithm to identify sociodemographic risk profiles for weekly dual-product use and weekly e-cigarette-only use.

**Results:**

Over 13% of adolescents used only e-cigarettes at least weekly, 3% engaged in weekly dual e-cigarette and cigarette use, and less than 0.5% used only cigarettes. Available spending money was a common predictor of dual-product and e-cigarette-only use. Gender diverse youth and youth with lower perceived family affluence were at higher risk for dual-product use, while white and multiethnic adolescents were at greater risk of e-cigarette-only use. Two high-risk profiles were identified for e-cigarette-only use and four high-risk profiles were identified for dual product use.

**Conclusions:**

This study used a novel modelling approach (CART) to identify combinations of sociodemographic characteristics that profile high-risk groups for exclusive e-cigarette and dual-product use. Unique risk profiles were identified, suggesting that e-cigarettes are attracting new demographics of adolescents who have not previously been considered as high-risk for traditional cigarette use.

## Background

E-cigarettes have overtaken cigarettes as the most popular nicotine product used by Canadian youth. Among a large sample of Ontario youth in grades 9-12, past 30-day e-cigarette use increased from 7.6% in 2013-14 to 25.7% in 2018-19, while corresponding cigarette use declined from 11.0% to 7.9%. [1] More recent nationally representative estimates from 2021-22 showed that 29% of grade 7-12 students had tried e-cigarettes, while only 14% had ever tried smoking cigarettes. [2] This decrease in cigarette smoking and corresponding increase in e-cigarette use suggests that youth risk-taking via nicotine product use is changing. The frequency with which adolescents use e-cigarettes and the rates of dual e-cigarette and cigarette use are two concerning patterns. Canadian estimates from 2019 suggest that 47.9% of youth aged 12-17 who use e-cigarettes do so at least weekly, and 23.8% do so daily. [3] The same study found that 5.3% of 15-17 year olds have used both e-cigarettes and cigarettes, with e-cigarette use preceding cigarette use in two-thirds of dual users. [3] Dual-product use is concerning because it may increase total nicotine exposure, leading to increased health risks and risk of nicotine dependence. [4, 5] Despite this, few Canadian studies have examined frequent dual product use.

As a contemporary form of nicotine product use, e-cigarette vaping may attract different sociodemographic groups than traditional cigarette smoking, which can have consequences for targeted public health initiatives. Previous studies have shown various similarities and differences in the sociodemographic risk profiles of e-cigarette and cigarette users. Prevalence of both e-cigarette and cigarette use has generally been higher among males and older adolescents [1, 3, 6]; however, recent estimates suggest a trend toward more similar rates of use among girls and boys. [2] Males are more likely to engage in exclusive e-cigarette use [7, 8] or poly-tobacco use [7, 9] while females are more likely to use cigarettes exclusively. [7] E-cigarette use is also more likely among youth of higher household income [3, 7] while dual-product use is more likely than exclusive e-cigarette use among youth of lower perceived socioeconomic status (SES). [8] Differences have also been observed by race and ethnicity, with e-cigarette use more common among white and Hispanic adolescents than among Black or Asian adolescents. [1, 7–9] Dual-product use also appears more likely among white adolescents than among Black, Hispanic, or other minority ethnicity adolescents. [7, 9] However, there remains a lack of population-level research into rates of e-cigarette and dual-product use among certain sociodemographic groups, in particular gender diverse youth and youth of Asian and Middle Eastern minority ethnicities.

One important limitation of previous studies [7–9] examining sociodemographic risk factors for e-cigarette and dual-product use is that the incremental risk of each sociodemographic characteristic is considered separately. In reality, various sociodemographic characteristics will have intersecting influences that need to be accounted for to properly portray risk profiles. The current study addresses this limitation by examining risk profiles through a classification and regression tree (CART) approach [10] that models complex interactions among risk factors using a tree structure. The objective of the current study is to identify and compare sociodemographic risk profiles for exclusive e-cigarette use and dual-product use among a large sample of Canadian youth. This study focuses on a measure of at least weekly use to identify adolescents at highest risk for problematic e-cigarette and/or conventional cigarette use.

## Methods

### Study design and participants

The current study was completed as part of the Contemporary Risk-taking by Canadian Youth (RISCY) study. RISCY uses a youth-informed approach involving a continual feedback cycle with youth advisory committees to explore how adolescent risk-taking is changing over time and to identify those who may be inequitably affected by risk-taking and its health consequences. The RISCY study brings together two of the largest Canadian youth health surveys: the Health Behaviour in School-aged Children (HBSC) study, and the Cannabis, Obesity, Mental health, Physical activity, Alcohol, Smoking, Sedentary behaviour (COMPASS) study, to incorporate new measures of risk-taking into Canadian surveillance initiatives. RISCY has received ethics clearance from Brock University (REB#22-315).

The COMPASS study, used here, is an ongoing, prospective cohort survey study of Canadian secondary school students in Ontario, Alberta, British Columbia, and Quebec. COMPASS uses purposive sampling to recruit whole-school samples. Ethics approval for COMPASS has been obtained from the University of Waterloo (ORE#30118), Brock University (REB#18-099), CIUSSS de la Capitale-Nationale–Université Laval (#MP-13-2017-1264), and all participating school boards. Informed consent was obtained from participants and from parents/guardians of children. Additional details about the COMPASS study are available in print [11] and online [12].

The current study uses student-level sociodemographic and substance use data from the 2021-22 wave of COMPASS study. The sample includes 50,189 students in grades 9-12 (secondary 3-5 in Quebec) from 167 schools, corresponding to a typical age range of 13-18 years old. The COMPASS student survey is an online, self-administered, anonymous questionnaire completed within a school-specific two-week period, with optional allocated class time. [13] Using active-information, passive-consent protocols, parents/guardians of all eligible students were sent study permission information via email and/or automated school phone system a minimum of two weeks prior to the survey start date, with the option to actively withdraw permission for their child(ren) to participate. Additionally, students could decline participation at any time prior to submitting survey responses. The participation rate in 2021-22 was 68.4%.

### E-cigarette and cigarette use measures

#### Cigarette use

Ever use of cigarettes was measured using the yes/no question, “Have you ever tried a cigarette, even just a few puffs?” and past 30-day use was measured using the question “On how many of the last 30 days did you smoke one or more cigarettes?”, with response options of “None”, “1 day”, “2 to 3 days”, “4 to 5 days”, “6 to 10 days”, “11 to 20 days”, “21 to 29 days”, and “30 days (every day)”. These measures align with frequency measures previously used in the Youth Smoking Survey [14]. Cigarette smoking self-report has been shown to be an accurate indicator of cigarette use prevalence for Canadian adolescents [15].

#### E-cigarette use

Ever use of e-cigarettes was measured using the yes/no question, “Have you ever tried a vape, also known as an e-cigarette? (e.g., JUUL, Vype, Suorin, Smok)” and past 30-day use was measured using the question “On how many of the last 30 days did you use a vape?”, with response options of “None”, “1 day”, “2 to 3 days”, “4 to 5 days”, “6 to 10 days”, “11 to 20 days”, “21 to 29 days”, and “30 days (every day)”. These measures were developed to align with frequency measures for cigarette smoking. E-cigarette use self-report has been shown to be a valid indicator of e-cigarette use in adolescents and young adults [16, 17].

#### Use frequency classifications

For e-cigarette and cigarette use separately, participants who responded “No” to the question on ever use were classified as “Never” users and those who indicated “Yes” to ever use but responded “None” to past 30-day use were classified as “Non-current” users. Among participants who indicated any past 30-day use, use on 1-3 days was classified as “Infrequent” while use on four or more days was classified as “Weekly”. The cut-off of four or more days was chosen based on sample size considerations for the low number of cigarette users in this study. This cut-off provides a sufficient sample of cigarette users to ensure model stability in prediction of dual product use while still differentiating potentially problematic use from one-off or very infrequent use.

## Sociodemographic measures

### Age

Age was measured using the question “How old are you today?”, with response options of “12 years or younger”, “13 years”, “14 years”, “15 years”, “16 years”, “17 years”, “18 years”, and “19 years or older”.

### Gender identity

Participants were asked to indicate both their biological sex and current gender. Sex was measured using the question “What sex were you assigned at birth?” with response options for “Female”, “Male”, and “I prefer not to say”. Gender was measured using the question “Which gender do you most identity with?” with response options for “Girl/Woman”, “Non-binary person”, “Two-spirit”, “Boy/Man”, “I describe my gender differently”, and “I prefer not to say”. Missing values were assigned to participants who selected “I prefer not to say”. Gender identity was classified using a two-step process based on World Health Organization recommendations [18]. Participants who answered “Female” and “Girl/Woman” were classified as cisgender girl, those who answered “Male” and “Boy/Man” were classified as cisgender boy, and those who answered other combinations of sex and gender were classified as gender diverse.

### Race/ethnicity

Participant racial/ethnic background was measured using the question “Which race category best describes you? (Mark all that apply)” with response options for “Black”, “East Asian”, “Latino”, “Middle Eastern”, “South Asian”, “Southeast Asian”, “White”, “Another category”, “I do not know”, and “I prefer not to say”. Participants selecting more than one option were classified as multiethnic. This measure aligns with the Canadian Institute for Health Information guidance on race-based data collection [19].

### Available spending money

Participants were asked two questions related to the amount and source of individual weekly spending money. Amount of spending money was measured using the question “About how much money do you usually get each week to spend on yourself or to save?” with response options of “Zero”, “$1 to $5”, “$6 to $10”, “$11 to $20”, “$21 to $40”, “$41 to $100”, “More than $100”, and “I do not know how much money I get each week”. Participants who responded “I do not know” were classified as missing for analysis purposes. Source of spending money was measured using the question “Where do you get money to spend on yourself or to save? (Mark all that apply)” with response options “I do not usually get any money to spend on myself or to save”, “My parents/guardians give me money (e.g., an allowance)”, “I get a paycheque from a job (working evenings or weekends at a restaurant, store, etc.)”, and “I get paid cash for occasional work (babysitting, mowing lawns, shovelling snow, etc.)”. Participants selecting more than one source option were classified as having multiple sources of spending money.

### Family financial affluence

Relative family financial affluence was measured using the question “Would you say that you and your family are more or less financially comfortable than the average student in your class?” with response options for “More comfortable”, “As comfortable”, and “Less comfortable”. Participants were also asked the yes/no question “In your house, do you have your own bedroom?”, which is a component of the Family Affluence Scale [20] and can indicate lower family financial affluence. This measure has not been validated as a standalone indicator of socioeconomic status but was included in the current study because it is also hypothesized as a potential indicator of available private space to engage in substance use. The complete Family Affluence Scale was not available in the 2021-22 COMPASS student survey.

### Analysis

Participants with missing data on e-cigarette or cigarette use (*n* = 3,523; 7.0% of sample) were excluded from the analysis, resulting in a final analytic sample of 46,666 students from 167 schools. A contingency table of e-cigarette and cigarette use was calculated to assess dual product use. Participants were classified according to their weekly (i.e., four or more times in the last 30 days) product use as either dual-product users (use of both e-cigarettes and cigarettes), e-cigarette-only users, cigarette-only users, or infrequent/non-users. Sociodemographic characteristics were reported for the total sample, as well as by product use classification. Separate t-tests (continuous variables) and chi-square tests (categorical variables) were used to calculate the statistical significance of difference in means and proportions between each user group relatively to infrequent/non-users. Missing values were excluded from tests. For categorical variables with more than two categories, chi-square test residuals were examined to explore which sociodemographic categories most contributed to the differences between user groups (residual tables not presented).

Separate classification trees were constructed using Classification and Regression Tree (CART) analysis [10] to identify sociodemographic risk profiles for weekly dual-product use and weekly e-cigarette-only use. The CART algorithm divides the sample into subgroups by iteratively choosing the sociodemographic variables and cut points that provide maximum separation between groups with respect to probability of e-cigarette or dual product use. Overviews of the CART method in the context of public health are available [21, 22]. A stable classification tree for cigarette-only use could not be constructed due to the small number of cigarette-only users. Infrequent/non-users were used as the reference group for all tree models. All covariates were included as predictors in each classification model and missing values were accounted for using surrogate splitting variables. [23] Due to class proportion imbalance in rates of weekly use, a weighted loss function proportional to the class imbalance was used to improve model sensitivity. The Gini index was used to measure node impurity for splitting, and tree depth was capped at four levels of splits to avoid over-complexity. Area under the receiver operating characteristic curve (AUC) was used as the criterion for final tree selection, with pruning performed to mitigate overfitting using 10-fold cross-validation to select the smallest tree having an AUC within one standard error of the maximum AUC (i.e., the “1-SE” rule [10]). Terminal nodes with weekly use probabilities higher than the root node were classified as “high-risk” groups. To perform the CART analysis, “rpart” [24] routine within the “caret” [25] package was used in R software version 4.3.0 [26].

## Results

### Rates of e-cigarette and cigarette use

Table 1 shows contingent rates of e-cigarette and cigarette use. Examining marginal product use, 42.0% of adolescents had ever tried e-cigarettes with 16.2% using at least weekly, while 19.9% of adolescents had ever tried cigarettes with only 3.4% using at least weekly. Examining dual product use, nearly all adolescents who used cigarettes also used e-cigarettes at an equal or greater frequency. Weekly use rates were 3.0% for dual-product use, 13.2% for e-cigarette-only use, and 0.4% for cigarette-only use.

**Table 1:** Rates of e-cigarette and cigarette use among grade 9-12 students in the 2021-22 COMPASS sample

*n (%)*	**Cigarette Use**				
*	Never	Non-Current	Infrequent (1-3 days)	Weekly (4-30 days)	**Total**
**E-Cigarette Use**					
Never	26,494 (56.8%)	439 (0.9%)	75 (0.2%)	63 (0.1%)	**27,071 (58.0%)**
Non-Current	6,041 (12.9%)	2,286 (4.9%)	128 (0.3%)	80 (0.2%)	**8,535 (18.3%)**
Infrequent (1-3 days)	2,212 (4.7%)	819 (1.8%)	363 (0.8%)	83 (0.2%)	**3,477 (7.5%)**
Weekly (4-30 days)	2,624 (5.6%)	2,561 (5.5%)	1,015 (2.2%)	1,383 (3.0%)	**7,583 (16.2%)**
**Total**	**37,371 (80.1%)**	**6,105 (13.1%)**	**1,581 (3.4%)**	**1,609 (3.4%)**	**46,666 (100.0%)**

^*^Frequency classifications based on ever and past 30-day use. Non-Current is defined as ever use but no use in past 30-days

### Sample sociodemographic characteristics by weekly product use

Table 2 shows sample sociodemographic characteristics. The sample comprised 48.9% cisgender girls, 45.6% cisgender boys, and 5.5% gender diverse adolescents, and the average age was 15.5 (SD 1.1). The sample was 68.4% white, 21.0% had no weekly spending money, and 63.9% considered themselves to have average relative family financial comfort.

**Table 2:** Sociodemographic characteristics of grade 9-12 students in the 2021-22 COMPASS sample by product use classification

**Sociodemographic Characteristic** *[n (%)]*	**Total** **Sample** *N* = 46666	**Dual-Product User** *N* = 1383	**E-cigarette-Only User** *N* = 6200			**Cigarette-Only User** *N* = 226		**Infrequent/** **Non-user** *N* = 38857	
**Age**^**1**^		(***)	(***)			(***)			
Mean (SD)	15.5 (1.1)	15.8 (1.4)	15.8 (1.1)			15.9 (1.5)		15.5 (1.1)	
[Missing]	23	2	2			0		19	
**Gender identity**^**2**^		(***)	(***)			(***)			
Cisgender Boy	20817 (45.6%)	544 (40.8%)	2346 (38.5%)			99 (46.5%)		17828 (46.9%)	
Cisgender Girl	22341 (48.9%)	529 (39.7%)	3472 (56.9%)			70 (32.9%)		18270 (48.1%)	
Gender Diverse	2503 (5.5%)	259 (19.4%)	279 (4.6%)			44 (20.7%)		1921 (5.1%)	
[Missing]	1005	51	103			13		838	
**Racial/ethnic background**^**2**^		(***)		(***)	(***)				
Another Category	3511 (7.7%)	158 (11.7%)	482 (7.9%)			59 (26.5%)		2812 (7.4%)	
Black	1769 (3.9%)	121 (9.0%)	145 (2.4%)			18 (8.1%)		1485 (3.9%)	
East Asian	1849 (4.0%)	21 (1.6%)	54 (0.9%)			8 (3.6%)		1766 (4.6%)	
Latino	1118 (2.4%)	36 (2.7%)	113 (1.9%)			11 (4.9%)		958 (2.5%)	
Middle Eastern	794 (1.7%)	36 (2.7%)	53 (0.9%)			6 (2.7%)		699 (1.8%)	
Multiethnic	3243 (7.1%)	156 (11.6%)	451 (7.4%)			29 (13.0%)		2607 (6.8%)	
South Asian	898 (2.0%)	8 (0.6%)	37 (0.6%)			1 (0.4%)		852 (2.2%)	
Southeast Asian	1302 (2.8%)	9 (0.7%)	51 (0.8%)			2 (0.9%)		1240 (3.3%)	
White	31315 (68.4%)	804 (59.6%)	4722 (77.3%)			89 (39.9%)		25700 (67.4%)	
[Missing]	867	34	92			3		738	
**Amount of Weekly Spending Money**^**2**^		(***)		(***)			(*)		
Zero	7962 (21.0%)	162 (13.5%)	455 (8.4%)			27 (13.9%)		7318 (23.4%)	
$1-5	1460 (3.8%)	44 (3.7%)	100 (1.9%)			10 (5.2%)		1306 (4.2%)	
$6-10	1764 (4.6%)	37 (3.1%)	142 (2.6%)			9 (4.6%)		1576 (5.0%)	
$11-20	3740 (9.8%)	81 (6.7%)	367 (6.8%)			13 (6.7%)		3279 (10.5%)	
$21-40	4071 (10.7%)	92 (7.6%)	601 (11.2%)			25 (12.9%)		3353 (10.7%)	
$41-100	5685 (15.0%)	175 (14.5%)	1004 (18.6%)			36 (18.6%)		4470 (14.3%)	
>$100	13311 (35.0%)	613 (50.9%)	2717 (50.4%)			74 (38.1%)		9907 (31.7%)	
[Missing]	8673	179	814			32		7648	
**Source of Weekly Spending Money**^**2**^		(***)		(***)			(.)		
None	9477 (20.4%)	224 (16.3%)	589 (9.6%)			41 (18.2%)		8623 (22.3%)	
Allowance	8894 (19.2%)	209 (15.2%)	820 (13.3%)			59 (26.2%)		7806 (20.2%)	
Occasional Work	2879 (6.2%)	95 (6.9%)	252 (4.1%)			16 (7.1%)		2516 (6.5%)	
Paycheque	20072 (43.3%)	686 (49.9%)	3780 (61.3%)			90 (40.0%)		15516 (40.2%)	
Multiple Sources	5077 (10.9%)	160 (11.6%)	725 (11.8%)			19 (8.4%)		4173 (10.8%)	
[Missing]	267	9	34			1		223	
**Relative Family Financial Comfort**^**2**^		(***)		(***)			(***)		
More comfortable	13064 (28.1%)	434 (31.6%)	1814 (29.3%)			70 (31.1%)		10746 (27.8%)	
As comfortable	29706 (63.9%)	676 (49.3%)	3711 (59.9%)			113 (50.2%)		25206 (65.2%)	
Less comfortable	3701 (8.0%)	262 (19.1%)	667 (10.8%)			42 (18.7%)		2730 (7.1%)	
[Missing]	195	11	8			1		175	
**Own Bedroom**^**2**^		(***)	(***)			(***)			
Yes	43671 (93.8%)	1196 (86.9%)	5980 (96.6%)			194 (86.6%)		36301 (93.6%)	
No	2902 (6.2%)	181 (13.1%)	208 (3.4%)			30 (13.4%)		2483 (6.4%)	
[Missing]	93	6	12			2		73	

1,2 – Statistical significance of differences in group means was assessed separately for each product use group relative to the infrequent/non-user group using a two-sided t-test (1) or chi-square test (2).

(*)*p* <.05

(**)*p*< .01

(***)p < .001

(.)*p* > .05

Table 2 shows sample characteristics across product use groups as well as statistical significance levels of differences in sample proportions relative to the infrequent/non-user group. Average age was similar across groups of weekly product users and approximately 0.3-0.4 years higher than the infrequent/non-user group. A disproportionately high percentage of dual-product and cigarette-only users were gender diverse relative to infrequent/non-users (19.4% and 20.7% vs. 5.1%). A higher proportion of e-cigarette-only users identified as white ethnicity (77.3% vs. 67.4%) and higher proportions of dual-product and cigarette-only use identified as Black, Latino, Middle Eastern, or multiethnic, relative to infrequent/non-users. Lower proportions of dual product, e-cigarette only, and cigarette only users identified as East Asian, South Asian, or Southeast Asian. A higher proportion of dual-product and e-cigarette only users had over $100 per week in available spending money (50.9% and 50.4% vs. 31.7%), primarily from a paycheque. A disproportionately high percentage of dual-product and cigarette-only users perceived their family to be relatively less financially comfortable (19.1% and 18.7% vs. 7.1%) and did not have their own bedroom (13.1% and 13.4% vs. 6.4%).

### Risk profiles of weekly dual-product users

**Fig. 1:**
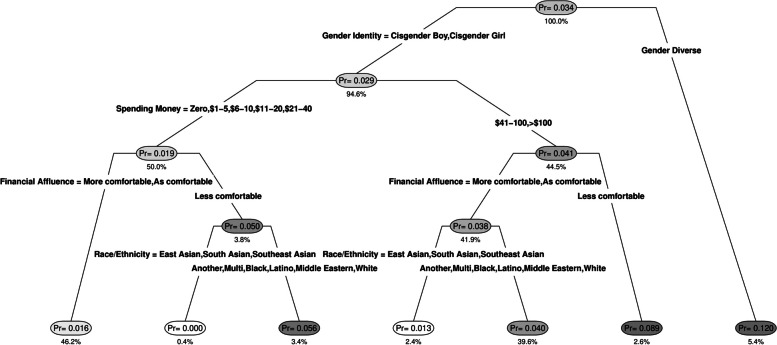
Classification tree* predicting weekly dual-product use of e-cigarettes and cigarettes vs. infrequent or non-use ^*^Pr = within-node probability of use; the percentage under the node refers to the percentage of the analytic sample contained within the node

Figure 1 shows the classification tree predicting weekly dual use of e-cigarettes and cigarettes. Gender identity emerged as a key differentiator, with probability of dual-product use four times higher in gender diverse adolescents than cisgender adolescents. Individual weekly spending money and family financial comfort emerged as opposing risk factors, with higher probability of dual-product use among those with higher individual spending money but relatively lower family financial comfort. Differences by race/ethnicity also emerged for some subgroups of cisgender adolescents, with much lower probability of dual-product use among East Asian, South Asian, and Southeast Asian adolescents.

Seven unique risk profiles were identified corresponding to the seven terminal tree nodes, and four groups were classified as “high-risk”, with probability of dual product use higher than the root node rate of 3.4%. The highest-risk group comprised gender diverse adolescents, who had a 12.0% probability of dual-product use. The second high-risk group comprised cisgender adolescents with over $40 per week in available spending money and relatively less family financial comfort, who had an 8.9% probability of dual-product use. The third and fourth high-risk groups both comprised cisgender adolescents of Black, Latino, Middle Eastern, multiethnic, white, or another ethnicity. Those with spending money under $40 per week and relatively less family financial comfort had a 5.6% probability of dual product use, while those with spending money over $40 per week but average or more family financial comfort had a 4.0% probability of dual-product use.

### Risk profiles of weekly e-cigarette-only users

**Fig. 2:**
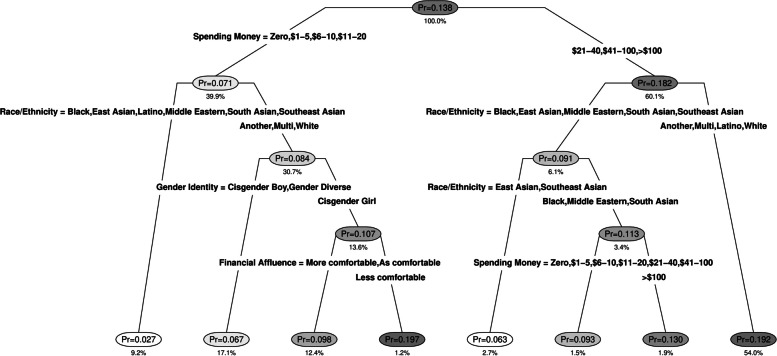
Classification tree* predicting weekly e-cigarette-only use vs. infrequent or non-use ^*^Pr = within-node probability of use; the percentage under the node refers to the percentage of the analytic sample contained within the node

Figure 2 shows the classification tree predicting weekly e-cigarette-only use. Available weekly spending money emerged as a key differentiator, with higher probability of e-cigarette-only use among those with over $20 per week in available spending money. Differences by ethnicity also emerged, with much higher probability of e-cigarette-only use among white, multiethnic, and Latino adolescents. Gender identity and family financial comfort also emerged as differentiators of use among subgroups of white and multiethnic adolescents with low spending money, with higher probability of use among cisgender girls with lower family financial comfort.

Eight unique risk profiles were identified corresponding to the eight terminal tree nodes, and two groups were classified as “high-risk” with probability of dual product use higher than the root node rate of 13.8%. The highest risk group comprised cisgender girls of white, multiethnic, or another ethnicity who had spending money under $20 per week and relatively low family financial comfort: the probability of e-cigarette-only use in this group was 19.7%. The second-highest risk group comprised adolescents of Latino, white, multiethnic, or another ethnicity with over $20 per week in spending money, who had a 19.2% probability of e-cigarette-only use.

## Discussion

This study examined differences in sociodemographic risk profiles for adolescents engaging in contemporary (i.e. e-cigarette vaping) and traditional (i.e. cigarette smoking) forms of nicotine product use. Nearly all cigarette users in this study also used e-cigarettes. Over 16% of youth in the sample used e-cigarettes at least weekly with 3% engaging in dual e-cigarette and cigarette use, while less than 0.5% of youth used only cigarettes. These findings are in line with past 30-day dual product use estimates of 5.3% in Canadian grade 7-12 students [3] and 2.7% to 8.9% (weighted average 5.3%) for grade 8-12 students in the United States [9]. While youth with available spending money had higher probabilities of both dual-product and exclusive e-cigarette use, risk profiles differed on other characteristics, suggesting that e-cigarettes are attracting different demographics of adolescents from those at high-risk for conventional cigarette use. Additionally, the sociodemographic risk factors identified for dual-product use but not e-cigarette-only use —namely, identifying as gender diverse and having lower family socioeconomic position— are consistent with risk factors traditionally associated with cigarette smoking [27–29], suggesting a shift among these traditional at-risk groups toward contemporary modes of use.

The largest high-risk group for exclusive e-cigarette use included white, Latino, and multiethnic adolescents who had over $20 in available weekly spending money. The CART analysis found higher probabilities of e-cigarette use among white and Hispanic adolescents compared to those of Black ethnicity, and this is consistent with other recent findings [7–9]; however, studies from the United States have found relatively lower rates of e-cigarette use among Hispanic adolescents compared to white students [7]. The other key differentiating factor for this risk group is available spending money, which has well-established associations to youth substance use [30–32]. Notably, the risk profiles differentiated on spending money rather than on family-level affluence. The increased risk associated with spending money could potentially be related to adolescents’ ability to purchase e-cigarettes rather than their overall socioeconomic status. Notably, this high-risk sociodemographic group comprised over half of the study sample and did not differentiate on gender or age, suggesting that e-cigarette use is widespread across many demographic groups who can access vaping devices. The results of the current study highlight the need for broad, universal strategies to limit e-cigarette availability and access across sociodemographic groups.

Four risk profiles were identified for youth at high risk of dual-product use. Gender diverse youth were the highest risk group, with rates of weekly dual-product use more than four times higher than cisgender adolescents. These findings are consistent with a recent review [27] showing higher rates of both e-cigarette and cigarette use among gender diverse youth, with evidence that these higher rates may be attributable to experienced gender minority stressors (e.g., discrimination, victimization) [27]. Gender diverse youth report stress relief and conforming to peer social norms as primary reasons for smoking and vaping [33]. Past population-level Canadian studies have not distinguished gender diverse youth in gender-stratified estimates of e-cigarette or cigarette use. The results of the current study highlight the need to represent gender diverse youth in national estimates that inform needs-based prevention initiatives. Notably, the elevated risk for gender diverse youth was specific to dual-product use as opposed to exclusive e-cigarette use. Given their unique stressors, tailored anti-tobacco campaigns may be more effective for gender diverse youth [33]. As an important caveat, this high-risk group comprised only 5.5% of the current sample, and so initiatives that target only this group are unlikely to significantly reduce population-level rates of e-cigarette and cigarette use; inclusive programming and multi-targeted initiatives are likely needed.

Cisgender adolescents in high-risk groups for dual product use had either high levels of individual spending money or lower relative family affluence, with the highest risk group having both attributes. The opposite directionality of association for these two SES proxy measures seems initially paradoxical. As previously discussed as a driver for e-cigarette-only use, higher individual spending money may increase risk of dual-product use through increased access and ability to purchase substances. In contrast, the findings regarding lower family affluence align with a previous study that found increased risk specifically for dual-product use compared to exclusive e-cigarette use among adolescents with lower family affluence [8]. This unique association between lower family affluence and dual-product use could suggest different influences behind decisions to use cigarettes and e-cigarettes. For example, the association between lower family affluence and youth cigarette use has been partially attributed to parents’ smoking behaviours [34]. Additionally, lower SES has been associated with greater exposure to cigarette advertising, while higher SES was associated with greater exposure to e-cigarette advertising among youth [35]. General tobacco prevention programming may benefit youth of lower relative family affluence; however, a better understanding of the drivers of dual-product use among this group is needed.

This study used a novel modelling approach to address a research gap in understanding adolescent risk profiles for problematic cigarette, e-cigarette, and dual use; however, several limitations are noteworthy. While this study included a large sample of Canadian youth, the sampling design was not representative and therefore any generalizations should be made with caution. While this study included a more comprehensive measure of gender identity than previous Canadian research, the sample required collapsing all gender diverse adolescents into one category. Also, no absolute measure of family-level SES (e.g., household income) was available. Not having one’s own bedroom is one indicator that can be associated with household material affluence [20], but this measure did not emerge as an important differentiator of risk in this study. Additionally, the measures of cigarette and e-cigarette use did not assess quantity of consumption, and the measure of e-cigarette use did not distinguish between nicotine-containing and nicotine-free products. The chosen frequency cut-off of four or more days in the past 30 days was selected to represent approximately weekly use, though it is not known if actual use was evenly distributed across weeks. This measure is also less stringent than the commonly used criterion for frequent use of 20+ days due to limitations in the sample size of cigarette smokers in this study. From a modelling standpoint, the CART algorithm does not account for the clustered nature of participants within schools, which could influence choice of split; however, modelling limitations for categorical outcomes prevented accounting for this clustered sampling design in the decision tree models. The resulting decision tree models also had modest fit statistics, with AUC values ranging from 0.63 to 0.67. CART analysis does not use statistical tests of determine if probabilities are statistically significantly different from each other, and therefore differences are understood as descriptive in nature rather than inferential. Thus, while the decision tree models were able to identify sociodemographic groups with varying probabilities of use, the included sociodemographic factors don’t fully explain differences between users and non-users. Future research should examine additional behavioural, interpersonal, and contextual drivers of use.

## Conclusions

This study used a novel modelling approach (CART) to identify combinations of sociodemographic characteristics that profile high-risk groups for exclusive e-cigarette and dual-product use. Most weekly users in this study exclusively used e-cigarettes, and available spending money was a key driver of e-cigarette and dual-product use across several sociodemographic groups. Nearly all traditional cigarette users also used e-cigarettes, with gender diverse and less affluent adolescents in high-risk groups for dual product use. The unique risk profiles identified for exclusive e-cigarette use and dual-product use suggest that new demographics of adolescents are at risk for problematic e-cigarette use.

## Data Availability

The datasets used in the current study are available upon successful completion of a COMPASS data usage application, available at https://uwaterloo.ca/compass-system/information-researchers

## Abbreviations

CART: classification and regression tree
COMPASS: Cannabis, Obesity, Mental health, Physical activity, Alcohol, Substance use, Sedentary behaviour
RISCY: Contemporary risk-taking in Canadian Youth
SES: Socioeconomic status

## References

[1] Cole AG, Aleyan S, Battista K, Leatherdale ST (2021). Trends in youth e-cigarette and cigarette use between 2013 and 2019: insights from repeat cross-sectional data from the COMPASS study. Can J Public Health..

[2] Summary of results for the Canadian Student Tobacco, Alcohol and Drugs Survey 2021. 2023 [cited 2023 Jul 4]. Available from: https://www.canada.ca/en/health-canada/services/canadian-student-tobacco-alcohol-drugs-survey/2021-2022-summary.html

[3] Rotterman M, Gilmour H. Correlates of vaping among adolescents in Canada. Ottawa, ON: Statistics Canada; 2022 [cited 2023 Sep 21]. (Health Reports). Available from: https://www.doi.org/10.25318/82-003-x202200700003-eng 10.25318/82-003-x202200700003-eng 35862070

[4] Wang JB, Olgin JE, Nah G, Vittinghoff E, Cataldo JK, Pletcher MJ (2018). Cigarette and e-cigarette dual use and risk of cardiopulmonary symptoms in the Health eHeart Study. PLOS ONE..

[5] Soneji S, Barrington-Trimis JL, Wills TA, Leventhal AM, Unger JB, Gibson LA (2017). Association Between Initial Use of e-Cigarettes and Subsequent Cigarette Smoking Among Adolescents and Young Adults: A Systematic Review and Meta-analysis. JAMA Pediatr..

[6] Craig W, Pickett W, King M. The health and wellbeing of Canadian adolescents: findings from the 2018 Health Behaviour in School-aged Children study. [Internet]. Public Health Agency of Canada; 2020 Jun p. 154p. Available from: https://www.canada.ca/en/public-health/services/publications/science-research-data/youth-findings-health-behaviour-school-aged-children-study.html

[7] Cho B, Hirschtick JL, Usidame B, Meza R, Mistry R, Land SR (2021). Sociodemographic Patterns of Exclusive, Dual, and Polytobacco Use Among U.S. High School Students: A Comparison of Three Nationally Representative Surveys. J Adolesc Health.

[8] Burnell K, Kwiatek SM (2021). Hoyle RH Are Exclusive e-Cigarette Users Unique? Comparing Predictors of Exclusive e-Cigarette Use with Traditional Tobacco Use and Dual Use among U.S. Adolescents. Subst Use Misuse..

[9] Usidame B, Hirschtick JL, Mattingly DT, Patel A, Patrick ME, Fleischer NL (2022). Sociodemographic Patterns of Exclusive and Dual Combustible Tobacco and E-Cigarette Use among US Adolescents—A Nationally Representative Study (2017–2020). Int J Environ Res Public Health..

[10] Breiman L (2017). Classification and Regression Trees.

[11] Leatherdale ST, Brown KS, Carson V, Childs RA, Dubin JA, Elliott SJ (2014). The COMPASS study: a longitudinal hierarchical research platform for evaluating natural experiments related to changes in school-level programs, policies and built environment resources. BMC Public Health..

[12] Shaping the direction of youth health | COMPASS System. [cited 2023 Nov 14]. Available from: https://uwaterloo.ca/compass-system/

[13] Reel B, Battista K, Leatherdale ST. COMPASS protocol changes and recruitment for online survey implementation during the COVID-19 pandemic. Waterloo, Ontario: University of Waterloo; 2020 Dec. (COMPASS Technical Report Series). Report No.: 7(2). Available from: https://uwaterloo.ca/compass-system/publications/compass-protocol-changes-and-recruitment-online-survey

[14] Elton-Marshall T, Leatherdale ST, Manske SR, Wong K, Ahmed R, Burkhalter R (2011). Research methods of the Youth Smoking Survey (YSS). Chronic Dis Inj Can..

[15] Wong SL, Shields M, Leatherdale S, Malaison E, Hammond D (2012). Assessment of validity of self-reported smoking status. Health Rep..

[16] Vogel EA, Prochaska JJ, Rubinstein ML (2020). Measuring e-cigarette addiction among adolescents. Tob Control..

[17] Doran N, Correa JB, Myers MG, Tully L (2020). Associations Between Self-Reported and Biological Measures of Nicotine Consumption Among Young Adult Nondaily Cigarette Smokers. Am J Addict..

[18] Költő A, Heinz A, Moreno-Maldonado C, Cosma A, Piper A, Saewyc EM, et al. Measuring sex and gender identity in a cross-national adolescent population survey: Perspectives of adolescent health experts from 44 countries. In: Excellence in Pediatrics - 12th Conference. United Kingdom: Taylor & Francis Group; 2020 [cited 2023 Nov 10]. Available from: https://orbilu.uni.lu/handle/10993/44926

[19] Canadian Institute for Health Information. Guidance on the Use of Standards for Race-Based and Indigenous Identity Data Collection and Health Reporting in Canada. Ottawa, ON: Canadian Institute for Health Information; 2022.

[20] Hartley JEK, Levin K, Currie C (2016). A new version of the HBSC Family Affluence Scale - FAS III: Scottish Qualitative Findings from the International FAS Development Study. Child Indic Res..

[21] Battista K, Diao L, Patte KA, Dubin JA, Leatherdale ST (2023). Examining the use of decision trees in population health surveillance: an application to youth mental health survey data in the COMPASS study. Health Promot Chronic Dis Prev Can Res Policy Pract..

[22] Lemon SC, Roy J, Clark MA, Friedmann PD, Rakowski W (2003). Classification and regression tree analysis in public health: Methodological review and comparison with logistic regression. Ann Behav Med..

[23] Therneau TM, Atkinson EJ. An introduction to recursive partitioning using the RPART routines. Mayo Foundation: Technical report; 2022.

[24] Therneau T, Atkinson B, Ripley B. rpart: Recursive Partitioning and Regression Trees. 2022 [cited 2023 Jun 15]. Available from: https://cran.r-project.org/web/packages/rpart/index.html

[25] Kuhn M, Wing J, Weston S, Williams A, Keefer C, Engelhardt A, et al. caret: Classification and Regression Training. 2023 [cited 2023 Jun 15]. Available from: https://cran.r-project.org/web/packages/caret/index.html

[26] R Core Team. R: A Language and Environment for Statistical Computing. Vienna, Austria: R Foundation for Statistical Computing; 2023. Available from: https://www.R-project.org/

[27] Fahey KML, Kovacek K, Abramovich A, Dermody SS (2023). Substance use prevalence, patterns, and correlates in transgender and gender diverse youth: A scoping review. Drug Alcohol Depend..

[28] Wheldon CW, Watson RJ, Fish JN, Gamarel K (2019). Cigarette Smoking Among Youth at the Intersection of Sexual Orientation and Gender Identity. LGBT Health..

[29] Hanson MD, Chen E (2007). Socioeconomic Status and Health Behaviors in Adolescence: A Review of the Literature. J Behav Med..

[30] Lozza E, Jarach CM, Sesini G, Marta E, Lugo A, Santoro E (2023). Should I give kids money? The role of pocket money on at-risk behaviors in Italian adolescents. Ann Ist Super Sanita..

[31] McCrystal P, Percy A, Higgins K (2007). The cost of drug use in adolescence: Young people, money and substance abuse. Drugs Educ Prev Policy..

[32] Zhang B, Cartmill C, Ferrence R (2008). The role of spending money and drinking alcohol in adolescent smoking. Addiction..

[33] Ma J, Kraus AJ, Owens C, Moskowitz DA, Birnholtz J, Macapagal K (2022). Perspectives on Cigarette Use, Vaping, and Antitobacco Campaigns Among Adolescent Sexual Minority Males and Gender Diverse Youth. LGBT Health..

[34] Soteriades ES, DiFranza JR (2003). Parent’s Socioeconomic Status, Adolescents’ Disposable Income, and Adolescents’ Smoking Status in Massachusetts. Am J Public Health..

[35] Simon P, Camenga D, Morean M, Kong G, Bold KW, Cavallo DA (2018). Socioeconomic Status (SES) and Adolescent E-cigarette Use: The Mediating Role of E-Cigarette Advertisement Exposure. Prev Med..

